# Development of genic-SSR markers by deep transcriptome sequencing in pigeonpea [*Cajanus cajan *(L.) Millspaugh]

**DOI:** 10.1186/1471-2229-11-17

**Published:** 2011-01-20

**Authors:** Sutapa Dutta, Giriraj Kumawat, Bikram P Singh, Deepak K Gupta, Sangeeta Singh, Vivek Dogra, Kishor Gaikwad, Tilak R Sharma, Ranjeet S Raje, Tapas K Bandhopadhya, Subhojit Datta, Mahendra N Singh, Fakrudin Bashasab, Pawan Kulwal, KB Wanjari, Rajeev K Varshney, Douglas R Cook, Nagendra K Singh

**Affiliations:** 1 National Research Centre on Plant Biotechnology, Indian Agricultural Research Institute, New Delhi 110 012, India; 2 Department of Molecular Biology and Biotechnology, University of Kalyani, Kalyani, WB 741235, India; 3 Division of Genetics, Indian Agricultural Research Institute, New Delhi, 110012, India; 4 Indian Institute of Pulses Research, Kanpur, UP 208024, India; 5 Institute of Agricultural Sciences, Banaras Hindu University, Varanasi, UP 221005, India; 6 University of Agricultural Sciences, Dharwad, Karnataka 580005, India; 7 Panjabrao Deshmukh Krishi Vidyapeeth, Krishinagar, Akola, Maharasthra 444 104, India; 8 International Crops Research Institute for the Semi-Arid Tropics, Patancheru, AP 502324, India; 9 Department of Plant Pathology, University of California, Davis, CA 95616-8680, USA

## Abstract

**Background:**

Pigeonpea [*Cajanus cajan *(L.) Millspaugh], one of the most important food legumes of semi-arid tropical and subtropical regions, has limited genomic resources, particularly expressed sequence based (genic) markers. We report a comprehensive set of validated genic simple sequence repeat (SSR) markers using deep transcriptome sequencing, and its application in genetic diversity analysis and mapping.

**Results:**

In this study, 43,324 transcriptome shotgun assembly unigene contigs were assembled from 1.696 million 454 GS-FLX sequence reads of separate pooled cDNA libraries prepared from leaf, root, stem and immature seed of two pigeonpea varieties, Asha and UPAS 120. A total of 3,771 genic-SSR loci, excluding homopolymeric and compound repeats, were identified; of which 2,877 PCR primer pairs were designed for marker development. Dinucleotide was the most common repeat motif with a frequency of 60.41%, followed by tri- (34.52%), hexa- (2.62%), tetra- (1.67%) and pentanucleotide (0.76%) repeat motifs. Primers were synthesized and tested for 772 of these loci with repeat lengths of ≥18 bp. Of these, 550 markers were validated for consistent amplification in eight diverse pigeonpea varieties; 71 were found to be polymorphic on agarose gel electrophoresis. Genetic diversity analysis was done on 22 pigeonpea varieties and eight wild species using 20 highly polymorphic genic-SSR markers. The number of alleles at these loci ranged from 4-10 and the polymorphism information content values ranged from 0.46 to 0.72. Neighbor-joining dendrogram showed distinct separation of the different groups of pigeonpea cultivars and wild species. Deep transcriptome sequencing of the two parental lines helped *in silico *identification of polymorphic genic-SSR loci to facilitate the rapid development of an intra-species reference genetic map, a subset of which was validated for expected allelic segregation in the reference mapping population.

**Conclusion:**

We developed 550 validated genic-SSR markers in pigeonpea using deep transcriptome sequencing. From these, 20 highly polymorphic markers were used to evaluate the genetic relationship among species of the genus *Cajanus*. A comprehensive set of genic-SSR markers was developed as an important genomic resource for diversity analysis and genetic mapping in pigeonpea.

## Background

Pigeonpea [*Cajanus cajan *(L.) Millspaugh] is an important food legume predominantly cultivated in the tropical and subtropical regions of Asia and Africa. It is a diploid (2n = 22), often cross-pollinated crop with a genome size of 858 Mbp [1]. Pigeonpea plays an important role in food and nutritional security because it is a rich source of protein, minerals and vitamins. Pigeonpea seeds are mainly consumed as split pea soups or 'dal' but a significant proportion is also eaten as green pea vegetable and as wholegrain preparations. In addition, pigeonpea leaves, seed husks and pods are used as animal feed, whereas the stem and branches are used as firewood. The world acreage of pigeonpea is 4.67 Mha with an annual production of 3.30 Mt. India is the largest producer and consumer of pigeonpea (local names 'arhar' and 'toor') with an annual production of 2.31 Mt, followed by Myanmar (0.60 Mt), Malawi (0.16 Mt) and Kenya (0.10 Mt) [2].

Knowledge of the genetic basis of yield, resistance to diseases and insect pests and abiotic stress tolerance are important factors for deciding the breeding strategies for genetic improvement of pigeonpea. However, in comparison to other economically important crops, relatively less effort has been invested in understanding the genetics of important agronomic traits of pigeonpea. Although there are ongoing efforts for pigeonpea improvement through conventional breeding, including hybrid technology, molecular breeding has a greater potential to accelerate the utilization of genetic resources in pigeonpea, especially among land races and related germplasm lines [3-8]. The availability of molecular markers that are tightly linked to important agronomic traits is a prerequisite for undertaking molecular breeding in plants. However, the genetic basis of most agronomic traits in pigeonpea has been worked out using conventional biometric techniques that have inherent limitations. The molecular basis of traits remains entirely unexplored and to date no molecular linkage map has been reported for pigeonpea [9,10]. This can be attributed to: (i) the low level of DNA polymorphism within the primary (cultivated) gene pool assessed by means of RAPD, RFLP, AFLP and recently by diversity array technologies (DArT) [11-15]; and (ii) a paucity of molecular markers available for genetic analysis in pigeonpea [16-20].

Simple sequence repeat (SSR) markers have the advantage of high abundance, random distribution within the genome, high polymorphism information content and co-dominant inheritance. However, genomic SSR markers developed from SSR-enriched genomic libraries or random genomic sequences are derived primarily from inter-genic DNA regions, and therefore have uncertain linkage to the transcribed regions of the genome. In contrast, genic-SSRs specifically target the transcribed region of the genome and have increased potential for linkage to loci that contribute to agronomic phenotypes. As a consequence, when polymorphic genic-SSRs are identified in high value breeding lines they can have considerable utility for marker assisted selection (MAS) [21]. Genic-SSR markers can also facilitate better cross-genome comparisons because they target protein-coding regions that are more likely to be conserved between related species [22]. Expressed sequence tags (ESTs) based on Sanger's sequencing technology have become increasingly abundant in public DNA databases and are being used for genetic analyses, comparative mapping, DNA fingerprinting, diversity analysis and evolutionary studies [23,24]; but only a limited number of pigeonpea Sanger ESTs are available in the public database [9].

We report the development of a large expressed sequence dataset based on 454 GS-FLX pyrosequencing of cDNA pools from two popular cultivars of pigeonpea, which are parents of the reference mapping population. We mined and validated a large set of genic-SSR markers and describe their application for understanding the genetic relationship among selected pigeonpea cultivars and wild *Cajanus *species. The dataset was also useful for *in silico *mining of polymorphic genic-SSR loci for the creation of an EST-based intra-species reference genetic map.

## Results

### Assembly of non-redundant transcriptome shotgun assembly (TSA) contigs of pigeonpea

**Figure 1:**
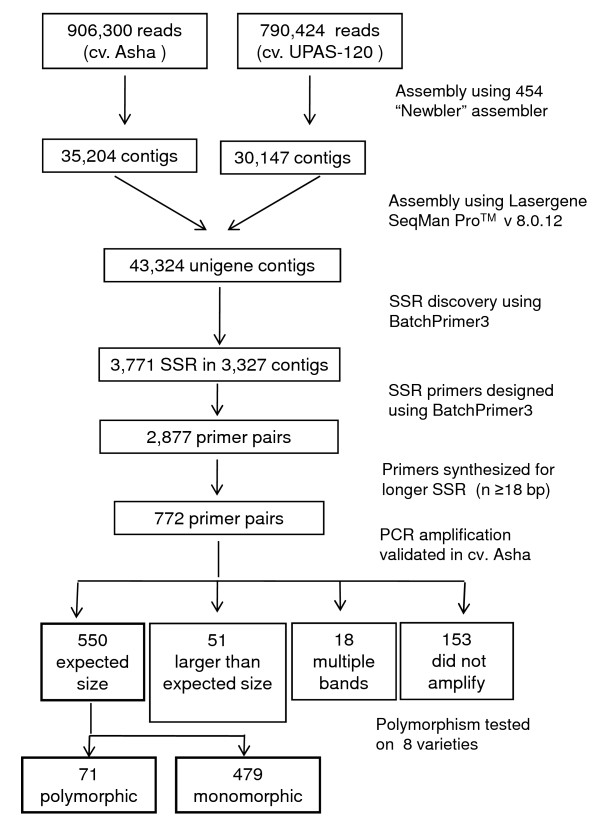
**Flow diagram of pigeonpea genic-SSR marker development**. Flow diagram illustrating development of genic-SSR markers in pigeonpea by deep transcriptome sequencing

Two runs of 454 GS-FLX pyrosequencing generated 1,696,724 high quality filtered expressed sequence reads from two separate cDNA library pools of widely adapted pigeonpea cultivars 'Asha' and 'UPAS 120'. In preparation for 454 sequencing, cDNAs were sheared stochastically to randomly represent all transcripts. Sequence data described in this paper can be found in the Sequence Read Archive (SRA) public database of the NCBI (Ac. No. SRP002556, SRP002557). The total dataset represents 566.6 Mbp of sequence with an average read length of 334 bp. These reads were first assembled separately into 35,204 TSA contigs for Asha (NCBI Ac. No. EZ647865- EZ683068) and 30,147 TSA contigs for UPAS 120 (NCBI Ac. No. EZ617718- EZ647864) using the 454 'Newbler' sequence assembler with average depths of coverage of 10.41 and 10.10, respectively (Table 1). To obtain a non-redundant set of unigene sequences, the total 65,351 TSA contigs from the two cultivars were assembled together using Lasergene SeqMan Pro™ Version 8.0.12 software into 43,324 unigene sequences with a total sequence length of 31.6 Mbp (Table 2). Of the 31.6 Mbp sequence, 21.9 Mbp was from 17,305 sequence contigs common to Asha and UPAS 120, 5.9 Mbp from 15,525 contigs unique to Asha, and 3.6 Mbp from 10,494 contigs unique to UPAS 120. This 31.6 Mbp of TSA sequence was 3.7% of the estimated 858 Mbp size of the pigeonpea genome and was used for *in silico *mining and validation of genic-SSR markers (Figure 1).

**Table 1:** Details of pigeonpea transcriptome shotgun sequence reads and their assembly into TSA contigs using 454-Newbler assembler

Variety	No. of sequence reads	Sequence length (bp)	Average read length (bp)	Average depth	Total no. of contigs	No. of bases in contigs (bp)
Asha	906,300	303,202,320	335	10.41	35,204	25,404,562
UPAS 120	790,424	263,411,375	333	10.10	30,147	22,824,365

Total	1,696,724	566,613,695	334	10.25	65,351	48,228,927

**Table 2:** Size distribution of the TSA contigs from two pigeonpea varieties generated using Newbler assembler and then aligned together using Lasergene SeqMan Pro™ software

Contig source	Contig size (bp)						
	
	1-100	101-200	201-300	301-400	400-500	>500	Total
Asha	65	2968	2835	4277	2624	2756	15525
UPAS 120	49	2403	2250	3052	1424	1316	10494
Common	0	223	297	536	738	15511	17305

Total	114	5594	5382	7865	4786	19583	43324

### Frequency distribution of different types of genic-SSR loci

A total of 3,771 SSR loci were identified in 3,327 TSA contigs, representing 7.6% of the total 43,324 unigene TSA contigs (Figure 1). This study did not include mononucleotide repeats, complex SSR or SSR loci with lengths less than 10 bp. Among the SSR containing contigs, 3,028 (91%) possessed single SSR loci, while 299 contigs (9%) had 2-4 SSR loci each. On an average there was one SSR locus for every 8.4 kbp of TSA unigene sequence, corresponding to one SSR for every 11.5 TSA unigene contigs. Dinucleotide was the most common repeat unit with a frequency of 60.41%, followed by tri- (34.52%), hexa- (2.62%), tetra- (1.67%) and pentanucleotide repeats (0.76%) (Additional file 1a). SSR loci with di- and trinucleotide repeats constituted 3,580 (95%) of the identified loci. The number of reiterations of a given repeat unit varied from 5 to 22 (Additional file 1b), and SSRs with five reiterations (the minimum limit set during the SSR marker discovery) were the most abundant. The frequency of a given SSR structure and the number of repeat units in it showed an inverse relationship (Additional file 1b). Hence, SSR loci with less than five repeats are expected to be even more abundant but were not included in the present investigation because they would not be useful in the study of detectable polymorphism [25]. Motifs showing more than 10 reiterations were rare with a frequency of <1% (Additional file 2). SSR markers with a length of 10 bp, the low end cut-off for SSR retention, were the most frequent (36%) followed by 15 bp (19%), 18 bp (12%) and 12 bp (11%) lengths; the longest SSR locus was of 66 bp (Additional file 1c). From the 3,771 genic-SSR sequences, 207 distinct repeat motifs were identified, (the 10 most frequent motifs are shown in Additional file 2). Dinucleotide repeat units TC/GA, AG/CT and TA/TA were the most abundant with frequencies of 17.20%, 16.67% and 9.41%, respectively. Among the trinucleotide repeat motifs, GAA/TTC and CTT/AAG were the most abundant with frequencies of 3.87% and 2.65%, respectively (Additional file 2).

### Development and validation of genic-SSR markers

PCR primers were designed from the unique sequences flanking 2,877 SSR loci identified in the TSA contigs for the development of genic-SSR markers and were designated ASSR1 to ASSR2877 (A= 'Arhar', Additional file 3). Primers could not be designed for the remaining 894 SSR loci because their flanking sequences were either too short or the nature of sequence did not fulfill the criteria for primer design using BatchPrimer3 v 1.0 software [26,27]. From the 2,877 SSR markers, 772 loci with n ≥ 18 bp including type I SSR markers (n ≥ 20 bp) were selected for primer synthesis and validation due to their high chance of showing polymorphism on agarose gel electrophoresis [25,28].

Of the 772 genic-SSR loci for which primers were synthesized, 550 yielded PCR amplicons of expected size and we designated these as "validated genic-SSR markers", as shown in Additional file 4. In addition, 51 primer pairs amplified larger than the expected size products, 18 primer pairs amplified multiple products (≥3 bands), and 153 primer pairs failed to amplify even when the annealing temperature was reduced by 7°C. All the 550 validated genic-SSR markers were scored for amplicon size polymorphism among eight pigeonpea varieties showing 71 (12.9%) polymorphic loci. Sixty-six of these polymorphic genic-SSR loci showed only two alleles each among the eight tested varieties; four loci possessed three alleles each, while one SSR locus (ASSR281) possessed five alleles. The PIC values of the 71 polymorphic genic-SSR markers ranged from 0.23 to 0.83 with an average of 0.38 (Table 3). Although a large proportion of the SSR loci was monomorphic in the eight pigeonpea varieties, some of these are likely to show polymorphism on analysis of a larger set of varieties. Use of more sensitive techniques for DNA fragment size analysis, e.g. polyacrylamide gel electrophoresis or capillary electrophoresis, is also expected to show a higher rate of polymorphism.

**Table 3:** Details of 71 genic-SSR loci showing polymorphism among 8 pigeonpea cultivars

**S. No**.	**Marker Id**.	**(SSR Motif)**_**n**_	Product size	No. of alleles	PIC value
1	ASSR1	(GA)_10_	100	2	0.47
2	ASSR3	(AGAAAG)_5_	145	2	0.47
3	ASSR5	(AAATT)_6_	130	2	0.36
4	ASSR8	(AGA)_9_	140	2	0.50
5	ASSR9	(AGA)_8_	150	2	0.23
6	ASSR11	(CTC)_7_	140	2	0.23
7	ASSR12	(AACAC)_6_	165	2	0.38
8	ASSR13	(ATTAG)_5_	160	2	0.37
9	ASSR15	(CAA)_8_	150	2	0.38
10	ASSR16	(GTT)_9_	150	2	0.23
11	ASSR17	(CCTTCT)_6_	180	2	0.38
12	ASSR19	(TGTTCA)_5_	160	2	0.38
13	ASSR20	(AT)_11_	140	2	0.23
14	ASSR23	(CCTTCT)_5_	150	2	0.47
15	ASSR48	(AAGAGG)_6_	150	2	0.30
16	ASSR66	(CT)12	180	2	0.44
17	ASSR70	(GGTAGA)_6_	170	2	0.45
18	ASSR77	(CT)_10_	140	2	0.41
19	ASSR93	(CATTTG)_5_	170	2	0.47
20	ASSR97	(ATGGAC)_8_	150	3	0.66
21	ASSR100	(GGT)_7_	150	2	0.23
22	ASSR108	(GAT)_7_	150	2	0.23
23	ASSR109	(GAA)10	140	2	0.38
24	ASSR120	(CTT)_7_	160	2	0.38
25	ASSR148	(CAA)_7_	110	2	0.50
26	ASSR153	(GAG)_8_	150	2	0.23
27	ASSR155	(TGGACA)_5_	130	2	0.23
28	ASSR169	(TCA)_7_	160	2	0.23
29	ASSR182	(ATT)_7_	220	2	0.38
30	ASSR205	(ATGAAG)_11_	170	2	0.38
31	ASSR206	(GTAATA)_6_	150	2	0.47
32	ASSR207	(ATCT)_5_	190	2	0.23
33	ASSR221	(TCG)_8_	165	2	0.23
34	ASSR228	(CTAAGG)_5_	140	3	0.53
35	ASSR229	(TAAGGG)_5_	160	3	0.53
36	ASSR230	(GAGCAT)_9_	170	2	0.38
37	ASSR236	(ACTAGC)_10_	230	2	0.23
38	ASSR237	(GGTGAA)_7_	180	2	0.23
39	ASSR247	(CACCAA)_6_	180	2	0.38
40	ASSR253	(CCCAAG)_6_	150	2	0.23
41	ASSR258	(CCATA)_5_	140	2	0.23
42	ASSR277	(TCCTGT)_5_	130	2	0.50
43	ASSR280	(TGGCAT)_5_	170	2	0.23
44	ASSR281	(CAAATG)_6_	220	5	0.83
45	ASSR286	(TGTTCA)_5_	160	2	0.38
46	ASSR293	(AGA)_7_	130	2	0.38
47	ASSR295	(ATA)_8_	140	2	0.38
48	ASSR297	(GCCACC)_5_	180	2	0.38
49	ASSR304	(GTT)_7_	110	2	0.50
50	ASSR317	(GAGCAT)_9_	150	2	0.47
51	ASSR352	(TTTAA)_6_	130	2	0.47
52	ASSR363	(GCATCA)_5_	190	2	0.50
53	ASSR366	(CGT)_8_	140	2	0.47
54	ASSR379	(TTCATG)_5_	140	2	0.47
55	ASSR380	(TTTC)_5_	170	2	0.23
56	ASSR390	(GAGCAA)_6_	190	2	0.50
57	ASSR408	(CAC)6	190	2	0.37
58	ASSR416	(TGA)6	210	2	0.37
59	ASSR427	(CT)9	170	2	0.37
60	ASSR495	(CT)9	200	2	0.50
61	ASSR610	(GTG)6	150	2	0.50
62	ASSR613	(CCA)6	150	2	0.21
63	ASSR895	(ATT)6	150	2	0.22
64	ASSR911	(AAT)6	140	2	0.47
65	ASSR980	(AAC)6	150	2	0.37
66	ASSR1193	(CA)9	180	2	0.47
67	ASSR1432	(TTC)6	140	2	0.47
68	ASSR1486	(TTG)6	140	2	0.37
69	ASSR1689	(AAT)6	140	2	0.37
70	ASSR1737	(TA)9	150	2	0.50
71	ASSR1848	(CAT)6	150	3	0.59

	**Average**			2.1	0.38

Annealing temperature for all 71 markers was 55°C. *Primer details and gene annotation is shown in Additional file 4.

The 550 SSR loci were searched against the non-redundant (nr) protein database of NCBI using BLASTX to assign functions to the TSA unigene sequences. This database includes all non-redundant GenBank CDS translations, PDB (Protein Data Bank), SwissProt, PIR (Protein Information Resource) and PRF (Protein Research Foundation), excluding environmental samples from Whole Genome Sequencing projects. The search output was used to categorize these expressed sequences into two classes: (i) putative known function, and (ii) unknown function, similar to that used for rice genes [29], except that there can be no hypothetical protein category here due to the transcriptomic origin of the sequences, hence matches with hypothetical protein annotations were also classified as unknown (Additional file 4). Putative known functions could be assigned to 297 (54%) sequences that showed a significant homology to reported proteins. The remaining 253 (46%) sequences were of unknown function, including 105 (19%) sequences which did not show a significant match in the database and therefore may encode proteins that are unique to the pigeonpea genome, or may correspond to an untranslated region (UTR) and/or diverged C-terminal coding region. Our analysis of the location of SSR markers within the TSA contigs revealed that among the 550 validated genic-SSR markers, 339 (61.6%) were located in the protein coding region, 87 (15.8%) in the 5'-UTR and 124 (22.5%) in the 3'-UTR (Additional file 4). Analysis of polymorphism among the three categories of genic-SSR loci revealed that those located in the 3'-UTR were the most polymorphic (23.4%), followed by 5'-UTR (12.6%) and coding sequences (9.1%). Further annotation of all the 43,324 TSA unigene contigs and single nucleotide polymorphism characterization between the reference varieties Asha and UPAS 120 is in progress.

### Assessment of genetic diversity among pigeonpea varieties and related species

**Figure 2:**
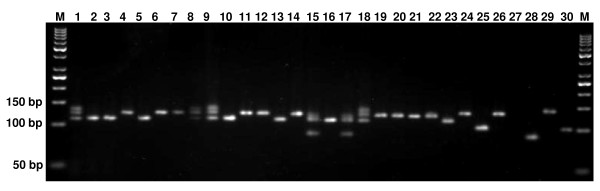
**Allelic variation for genic-SSR marker ASSR-277 among pigeonpea genotypes**. Agarose gel showing allelic variation among 30 pigeonpea genotypes with genic-SSR marker ASSR-277: 1 Asha, 2 UPAS 120, 3 HDM04-1, 4 Pusa dwarf, 5 H2004, 6 Bahar, 7 Maruti, 8 TTB7, 9 Pusa 992, 10 PS-971, 11 PS-956, 12 Pusa-9, 13 JA-4, 14 Kudarat, 15 PCMF40, 16 PCMF43-7, 17 PCMF39-1, 18 GT288A, 19 GTR-9, 20 GTR-11, 21 ICPA2089A, 22 ICPR2438, 23 *R. aurea*, 24 *C. platycarpus *(1), 25 *C. cajanifolius, 26 C. platycarpus *(2), 27 *R. braoteaca, 28 C. sericea, 29 C. albicans 30 C. lineatus*. M-50 bp DNA ladder

**Figure 3:**
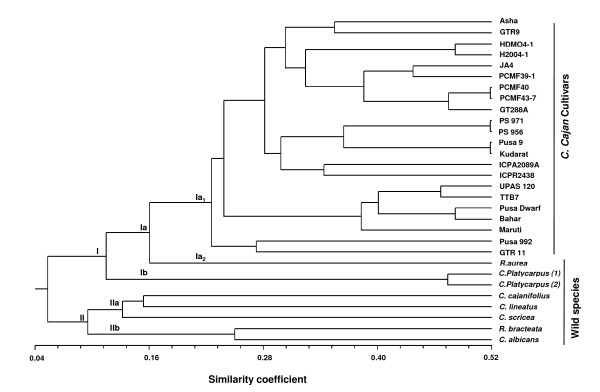
**Phylogenic relationship among pigeonpea varieties and wild species based on genic-SSR markers**. Dendrogram showing phylogenetic relationship among 22 *Cajanus cajan *varieties and 8 wild species generated from 20 genic-SSR markers. Scale at the bottom of the dendrogram indicates the level of similarity between the genotypes.

The 20 highly polymorphic genic-SSR markers designed in this study were used to assess the genetic diversity in a set of 30 genotypes representing diverse cultivated genotypes, wild species of *Cajanus *and inter-specific derivatives (Figure 2, Additional file 5). In total, 125 different DNA fragments with an average of 6.25 alleles per locus were amplified among the 30 genotypes. The number of alleles per SSR marker ranged from 4 for ASSR66 to 10 for ASSR3, whereas the PIC values ranged from 0.46 to 0.72 with an average of 0.63 per marker. As expected, a higher average number of alleles and PIC values were observed for the wild species (4.1 alleles per locus and 0.72 PIC value) compared to those for *C. cajan *cultivars (3.75 alleles per locus and 0.49 PIC value) (Table 4). Jaccard's similarity coefficients were calculated for pair-wise combinations of all the genotypes and a dendrogram was constructed to resolve the members of the primary, secondary and tertiary gene pools in the two main groups (Figure 3). Cluster I corresponded to the primary gene pool, including all the *C. cajan *cultivars in sub-cluster Ia_1_, while sub-cluster Ia_2 _was represented by a single entry *Rhynchosia aurea*. Cluster Ib included two genotypes of *C. platycarpus*, suggesting that it is closer to *C. cajan *than *C. cajanifolius*. Intra sub-cluster similarity in Cluster I ranged from 16.5% to 52%. Cluster II included the remaining five wild species of the secondary and tertiary gene pool (Figure 3). Cluster II was divided into two sub-clusters IIa and IIb, at a cut-off similarity index of 26%. The three wild species, namely *R. aurea, C. platycarpus *1 and 2, showed close relatedness to *C. cajan *cultivars but were in the tertiary gene pool due to low crossability with cultivated pigeonpea. Among the *C. cajan *cultivars, three pairs- PCMF40/PCMF43-7, Pusa 9/Kudarat and PS 971/PS 956 showed the highest similarity (52%).

**Table 4:** Details of 20 highly polymorphic pigeonpea genic-SSR markers, including a range of allele sizes, number of alleles and PIC values among 30 genotypes

**Sr. No**.	**Marker Id**.	Allele size (bp)	Number of alleles			PIC Value		
			Cultivars	Wild	Total	Cultivars	Wild	Total
1.	ASSR1	75-120	3	4	7	0.46	0.67	0.65
2.	ASSR3	125-150	3	3	4	0.43	0.85	0.69
3.	ASSR8	130-150	5	4	5	0.68	0.72	0.69
4.	ASSR23	130-170	4	4	5	0.40	0.55	0.62
5.	ASSR66	170-210	2	6	8	0.35	0.82	0.68
6.	ASSR70	150-190	4	3	7	0.23	0.78	0.48
7.	ASSR93	140-170	4	4	6	0.48	0.50	0.56
8.	ASSR97	90-150	3	7	10	0.29	0.86	0.56
9.	ASSR148	90-120	5	3	5	0.59	0.82	0.71
10.	ASSR206	115-155	2	2	6	0.63	0.77	0.69
11.	ASSR228	130-150	5	2	5	0.68	0.83	0.71
12.	ASSR277	90-145	2	7	8	0.30	0.61	0.53
13.	ASSR281	210-245	5	3	5	0.69	0.67	0.72
14.	ASSR304	90-120	6	6	9	0.35	0.82	0.64
15.	ASSR317	13-170	5	5	8	0.65	0.85	0.72
16.	ASSR352	110-145	3	3	5	0.77	0.69	0.65
17.	ASSR363	170-195	4	5	5	0.48	0.78	0.66
18.	ASSR366	135-150	3	5	6	0.33	0.63	0.52
19.	ASSR379	120-140	4	2	5	0.73	0.47	0.72
20.	ASSR390	160-195	3	4	6	0.41	0.72	0.46

	**Average**		**3.75**	**4.1**	**6.25**	**0.4965**	**0.7205**	**0.633**

### *In silico *analysis of SSR polymorphism between Asha and UPAS 120

**Figure 4:**
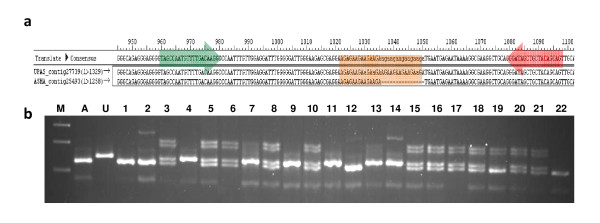
**Wet laboratory validation of *in silico *identified genic-SSR length polymorphism between pigeonpea parental lines**. Pigeonpea genic-SSR locus ASSR-8 showing: a. *in silco *polymorphism between the aligned TSA contigs of parental lines Asha (A) and UPAS 120 (U), b. agarose gel analysis of segregation of the ASSR-8 alleles in F_2 _population. Positions of flanking primers and the polymorphic SSR sequence are highlighted.

One aim of the present investigation was *in silico *identification of SSR polymorphism between pigeonpea varieties Asha and UPAS 120 for the development of an EST-based intra-species reference genetic map. TSA contigs were first assembled separately for Asha and UPAS 120 using the 454-Newbler assembler and then aligned together using Lasergene SeqMan Pro™ Version 8.0.12 software to obtain the SSR size differences between Asha and UPAS 120 varieties. A total of 1,484 SSR loci were present in the 17,305 TSA contigs common to Asha and UPAS 120. Only 318 of these loci were type I SSR (n ≥ 20 bp) of which 47 were polymorphic between Asha and UPAS 120 with size differences of 2-15 bp based on the *in silico *alignments. Further, only 24 of these loci showed allelic size differences of ≥4 bp, which is considered amenable for analysis on gel electrophoresis. For wet laboratory validation of polymorphism we chose these 24 SSR loci and designated them as ASSR1 to ASSR24. Four of the markers (ASSR4, ASSR6, ASSR18, ASSR22) did not amplify any PCR product, one marker (ASSR21) showed a larger than expected product size, while nine markers amplified but did not show distinct polymorphism on agarose gel electrophoresis perhaps due to small product size difference (average 4.8 bp difference), or actual lack of polymorphism. The remaining 10 primers (ASSR1, 3, 8, 9, 12, 13, 15, 17, 19, 23) showed distinct size polymorphism between Asha and UPAS 120 as expected from the *in silico *analysis (average 7 bp difference). Figure 4 presents such an example with ASSR8, where agarose gel electrophoresis confirmed the expected 15 bp size difference between Asha and UPAS120 and a Mendelian segregation ratio of 1:2:1 in the F_2 _mapping population derived from the cross between Asha and UPAS 120. Thus, 40% of the 24 *in silico *identified polymorphic SSR loci were validated successfully by wet laboratory analysis.

## Discussion

Conventional breeding of pigeonpea has continued entirely without the aid of molecular methods and made limited use of germplasm resources, resulting in a very narrow genetic base in the domesticated species. As a consequence, pigeonpea genetic improvement programs have made relatively little progress in addressing the primary constraints to crop production, which include a range of abiotic (e.g. drought, salinity and water-logging) and biotic (e.g. *Fusarium *wilt, sterility mosaic disease and pod boring insect *Helicoverpa armigera*) stresses. With the advent of next generation sequencing technologies several crop legumes have recently been subjected to intensive analyses, making marker-assisted breeding a reality [30]. Margulis *et al. *[31] demonstrated a 100-fold sequencing capability with 454 GS-FLX pyrosequencing but with relatively lower accuracy at the homopolymer positions than Sanger-based capillary electrophoresis sequencing. We used 454 GS-FLX pyrosequencing to develop an extensive collection of expressed sequence reads from two parental pigeonpea cultivars and mined and validated a comprehensive set of genic-SSR markers.

A total of 1.696 million high quality sequence reads were assembled to generate 43,324 TSA unigene contigs, which together represented a large fraction of the pigeonpea transcriptome and helped develop a comprehensive set of genic-SSR markers. Application of a subset of these markers in pigeonpea was sufficient to assess the genetic diversity among cultivars and position domesticated accessions relative to related species and genera. These markers represent a significant addition to the limited set of genic-SSR markers available in pigeonpea [16-20].

Only 81.8% of the SSR-containing unigene sequences showed significant hits in the NCBI non-redundant protein database. This may be due to: (i) EST fragments sequenced directly instead of after cloning; this leads to truly random sequencing of all the expressed genes that may facilitate the discovery of new rare transcripts as evidenced by Emrich *et al. *[32]; or (ii) unique contigs being part of a consensus sequence representing 3'-UTRs; C-termini or 3' sequences which are often less conserved than other transcript regions [33].

The deep transcriptome sequence data allowed the discovery of a set of 3,771 perfect SSR loci of ≥10 bp length in pigeonpea. About 7.6% of the pigeonpea TSA unigene contigs possessed at least one SSR- similar to previously reported SSR prevalence in the ESTs of wheat (7.41%), higher than grapes (2.5%), barley (2.8%) and flax (3.5%), but lower than coffee (18.5%) [22,34-37]. The genic-SSR frequency also depended on the parameters used in exploring SSR markers, e.g. the repeat length and number of repeat unit thresholds. The abundance of genic-SSR (kbp/SSR) in pigeonpea was 8.4 compared to 3.4 in rice, 5.4 in wheat, 7.4 in soybean, 11.1 in tomato, 14.0 in *Arabidopsis*, and 20.0 in cotton [37,38]. Differences in genic-SSR abundance could be partly due to the size of the EST unigene assembly dataset and use of different search criteria and data mining tools [21]. Frequency distribution of EST-SSR motifs in our study was not comparable with earlier work on pigeonpea by Raju *et al. *[20] because we developed TSA unigene contigs using FLX-454 sequencing, instead of Sanger sequencing. The number of SSRs identified in the present study was 3,771 from 43,324 unigene sequences, whereas Raju *et al. *described 3,583 SSRs from only 5,508 unigenes. The main reason for the overestimation of SSR frequency by Raju *et al. *is the inclusion of compound SSRs and homopolymers which are the most frequent repeats.

Dinucleotide SSR loci were most frequent in the pigeonpea TSA contigs analyzed here, representing 60.41% of the SSR loci identified, i.e. about double that of the trinucleotide SSR loci (34.52%), the second most abundant motifs. This was in agreement with the genic-SSR distribution reported in peach, pumpkin, spruce, coffee and kiwifruit, where dinucleotide repeats are most frequent [39-42]. However, this is in contrast to a number of earlier reports showing trinucleotides as the most abundant class of SSR loci in ESTs [22,35,43-47]. A possible explanation for the high frequency of dinucleotide SSR loci in pigeonpea TSA is that these include large amounts of information representing UTRs due to deep transcriptome sequencing. Yu *et al. *[48] reported 19% of dinucleotide repeats in the coding region and 81% in the 5'- and 3'-UTRs, whereas 74% of the trinucleotide repeats were in the coding regions and only 26% in UTRs. Among the 550 validated SSR loci with n≥18 bp in the present study, only 97 (17.6%) were dinucleotide repeats and 56 were in the UTR. Most of the dinucleotide SSR loci showed a smaller size range of 10-12 bp (Additional file 2). Our study also showed that the overall proportion of polymorphic SSR markers was much higher in UTRs compared to the coding region- there were 40 polymorphic SSR markers in UTRs (11 in 5'-UTR and 29 SSR in 3'-UTR), whereas only 31 were polymorphic in the coding region, despite 61.7% of all amplified SSR markers being located in the coding region. This is due to the tendency of sequence conservation in the coding regions.

Only 80% of the 772 tested SSR primers amplified the target pigeonpea genomic DNA. The success rate is comparable to barley, where 67-70% of the primers amplified [43,34], but higher than sugarcane (48.5%) and lower than flax (92.2%) [35,49]. A possible explanation for the lack of amplification could be flanking primers extending across a splice site with a large intron or chimeric cDNA contigs [34]. Although the majority (519 numbers) of the designed SSR markers amplified a single expected product size at the annealing temperature of 55°C, we optimized the annealing temperature of 31 additional primers to maximize the availability of genic-SSR markers for pigeonpea.

Generally genic-SSR markers show a lower level of polymorphism than genomic-SSR markers [22,50-52], but in this study the use of type I genic-SSR markers showed a high level of polymorphism. Previous diversity studies with pigeonpea species using genomic-SSR markers reported an average of 3.1-4.9 alleles per locus with average PIC values of 0.41-0.52 [15,17-19]. An earlier study with genic-SSR markers in pigeonpea reported the average number of alleles per marker as 4 and an average PIC value of 0.40 [20]. We observed a higher average of 6.25 alleles per locus and an average PIC value of 0.63 by using type I genic-SSR markers. The possible reasons are: (i) choice of 20 highly polymorphic SSR markers for the diversity assessment on 30 genotypes after initial testing of 550 markers on eight varieties; (ii) higher depth of coverage generated by the 454 GS-FLX sequencing technology that produced larger sequence contigs including UTRs which are more polymorphic; (iii) use of a diverse genotype set including interspecific derivatives and wild *Cajanus *species for diversity assessment. Contrary to other plant species where dinucleotide repeats showed high polymorphism [33,43,46], hexanucleotide repeats were highly polymorphic (38.57%) in pigeonpea genic-SSR markers, followed by pentanucleotides (29.14%) and trinucleotides (15.25%). Larger repeats have been linked to a higher degree of polymorphism in earlier studies [25,53]; we also found the maximum polymorphism with 40-50 bp SSR length on agarose gel electrophoresis.

On the basis of SSR polymorphism, cluster analysis and earlier diversity studies involving RFLP, AFLP, RAPD, SSR and DArT markers, it is concluded that genetic diversity in the pigeonpea gene pool is very low [11-20]. The genic-SSR markers reported here open up new opportunities to assess the genotypic diversity in the pigeonpea germplasm. Most of the earlier reported SSR markers in pigeonpea are of genomic origin except for 84 genic-SSR markers reported recently [20]. This study is the first report a comprehensive set of genic-SSR markers for pigeonpea.

Wild species of crop plants are placed in different gene pools based on their crossability with the cultivated species. Closely related and easily crossable species are placed in the primary or secondary gene pools, whereas species which are distantly related and are incompatible with the cultivated species, are placed in a tertiary gene pool. Species in the primary and secondary gene pools can be readily utilized for varietal improvement. In this study, the "unweighted pair group method with arithmetic mean" (UPGMA)-based cluster analysis grouped the genotypes according to their gene pool. The variability within the *C. cajan *cultivars which easily cross-hybridize among themselves formed the primary gene pool (Cluster I), whereas four species that have poor crossability with the *C. cajan *formed the secondary gene pool (Cluster II). Sub cluster Ib (*C. platycarpus *1 and 2) and sub-sub cluster Ia_2 _(*R. aurea*) included species from the tertiary gene pool even though they were closely related to *C. cajan *based on the marker analysis. The varieties of *C. cajan *showed different levels of similarity, e.g. PCMF 40 and PCMF 43-7, both inter-specific derivatives belonging to the short maturity group, shared 52% similarity between them and 40% similarity with PCMF 39-1 having similar pedigree. Likewise, short duration variety pair (PS-971/PS-956) and long duration variety pair (Pusa-9/Kudarat) were closer to each other than varieties belonging to different maturity groups. Genotypes of secondary and tertiary gene pools clustered separately into two sub-clusters. *R. aurea *and *C. platycarpus *belong to the tertiary gene pool due to poor crossability with the cultivated pigeonpea, but they showed genetic similarities with *C. cajan*. These results are also supported by Raju *et al. *[20] who used 15 EST-SSRs to study the genetic diversity of 32 cultivars and eight accessions of two *Cajanus *species *C. platycarpus *and *C. scarabaeoides*. Earlier, a close relationship was reported between *C. cajanifolius *and *C. cajan *using genomic SSR markers [17,19], but our study based on genic-SSR markers showed that *C. cajanifolius *is more distant to *C. cajan *compared to *C. platycarpus*. UPAS 120, TTB 7, Pusa Dwarf and Bahar genotypes belonging to different maturity groups were part of a single cluster. Genotypes of secondary and tertiary gene pools clustered separately in two sub-clusters, but *R. bracteata *which belongs to the tertiary gene pool based on the crossability criteria clustered with genotypes of the secondary gene pool. The closeness between *Cajanus *and *Rhynchosia *is also supported by morphological and genetic evidence, i.e. the presence of strophiole, an important characteristic used to distinguish between the genera. Seeds of *Cajanus *and *Rhynchosia *are generally described without strophioles. Various species of *Rhynchosia*, even though genetically closer to *Cajanas*, fail to produce hybrids because of reproductive barriers, and therefore *Rhynchosia *and *Cajanus *are classified as separate genera. High resolution mapping of these genotypes using a large number of genomic markers for diversity analysis may provide different results because genic-SSRs represent the transcribed portion of the genome, while the repetitive heterochromatin portion of the genome plays a major role in the evolution of species [54].

This is the first report of development and validation of a comprehensive set of genic-SSR markers in pigeonpea by deep transcriptome sequencing using next generation sequencing technology. A set of 2,877 genic-SSR markers was developed, and 550 SSR markers from this were validated for robust amplification in eight pigeonpea varieties, that will be useful for diversity analysis as well as mapping and tagging of genes and quantitative trait loci for economically important traits in pigeonpea.

## Conclusions

A dataset of 43,324 TSA unigene contigs derived from 1.69 million 454 GS-FLX sequence reads of two pigeonpea varieties was produced. A comprehensive set of 2,877 genic-SSR markers was developed and 550 of these were validated for amplification and polymorphism, which will be useful for the development of molecular maps based on genic markers. Of the 550, 20 highly polymorphic markers identified all the individuals of a set of 30 genotypes including cultivars and wild species. Due to conservation of genic sequences these markers have a higher chance of transferability across species, compared to genomic SSR markers which show high polymorphism but are less conserved between species. A combination of these genic-SSR markers, single nucleotide polymorphism markers being mined from the TSA contigs assembled in this study and genomic SSR markers developed in other laboratories will be a powerful resource for molecular taxonomic studies and construction of a reference molecular map of the pigeonpea genome. Since genic-SSR markers belong to the gene-rich regions of the genome, some of these can be exploited for use in marker-assisted breeding of pigeonpea. Therefore, the set of genic-SSR markers developed here is a promising genomic resource.

## Methods

### Plant materials

Root, leaf, stem and immature seeds from two pigeonpea varieties, namely Asha and UPAS 120, were used for RNA extraction and transcriptome sequencing. The 30 genotypes used for validation of SSR markers and diversity analysis included members of primary (20 cultivars of *C. cajan*), secondary (*C. albicans, C. cajanifolius, C. lineatus, C. sericeus*) and tertiary (*C. platycarpus*, *R. aurea, R. bracteata*) gene pools. The genotypes were originally obtained from IARI, New Delhi, ICRISAT Hyderabad, IIPR Kanpur, CCSHAU Meerut, JNKVV Jabalpur, GAU S.K. Nagar, and maintained at the Indian Agricultural Research Institute, New Delhi (Additional file 5).

### RNA extraction and cDNA sequencing

Plant RNA was isolated using the modified CTAB method [55]. One gram of frozen leaf, root, stem or immature seed tissue was separately ground in liquid nitrogen and mixed with 15 ml of extraction buffer (100 mM Tris-HCL (pH 8), 2% CTAB, 30 mM EDTA, 2 M NaCl, 0.05% spermidine, 2% polyvinylpolypyrrolidinone (PVP) and 2% 2-mercaptoethanol. The homogenate was incubated at 65°C for 10 min and extracted with chloroform-isoamyl alcohol (24:1), and RNA precipitated with 12 M LiCl. After washing with 70% ethanol the RNA pellet was dissolved in diethylpyrocaronate (DEPC) treated water. Equimolar concentrations of extracted RNA from the four different tissues of each variety were mixed to create two RNA pools and sent to Roche for 454 GS-FLX sequencing.

### Development of genic-SSR markers and *in silico *analysis of parental polymorphism

Expressed sequence reads were generated by deep transcriptome sequencing from two sets of normalized cDNA libraries. High quality filtered sequence reads were obtained by 454 GS-FLX sequencing, and sequence contigs were generated for the two varieties separately by *de novo *assembly using 454 'Newbler' assembler. Sequence data for *C. cajan *Short Read Archive (SRA) described in this paper can be found in the public database (Ac. no. SRP002556, SRP002557). A non-redundant set of unigene sequences was created by further alignments of the Newbler contigs from the two varieties using Lasergene SeqMan Pro™ Version 8.0.12 assembler with default parameters to develop 43,324 unigene contigs. This unigene set was used for mining genic-SSR markers and primer design using BatchPrimer3 v1.0 software [26,27]. In this study, the SSR loci containing perfect repeat units of 2-6 nucleotides only were considered. The minimum SSR length criteria were defined as five reiterations for each repeat unit. Mononucleotide repeats and complex SSR types were excluded from the study.

The parameters for designing primers from the SSR flanking sequences were: primer length range of 20-25 bases with an optimum of 22 bases; PCR product size range of 100-200 bp; optimum annealing temperature of 50-60°C; GC content of 40-60% with an optimum of 50%; the specified number of consecutive Gs and Cs at the 3' end of both primers was one. Other parameters were at the default setting of BatchPrimer3 v1.0 [26].

We also performed *in silico *analysis of parental polymorphism for SSR loci present in the 17,305 TSA contigs common to Asha and UPAS 120. Type I SSR loci with n≥20 bp were targeted and pair-wise alignment of these contigs was inspected manually to identify SSRs with a minimum size difference of 4 bp between Asha and UPAS 120. Primers were synthesized for the 24 SSR loci with size difference of ≥4 bp for validation by PCR amplification and agarose gel electrophoresis as described below.

### Plant DNA extraction, genotyping and annotation of gene function

Genomic DNA was isolated from leaf samples of 30 genotypes (Additional file-2) according to the CTAB method [56], quantified by UV_260 _absorbance and adjusted to a final concentration of 30 ng/μl. All the 772 genic-SSR loci with SSR lengths of 18 bp or longer were first tested for amplification using genomic DNA from Asha for optimization of the annealing temperature. The PCR reactions were performed using PTC225 Gradient Cycler (MJ Research). Each PCR reaction consisted of 1.5 μl of 10x reaction buffer, 0.20 μl of 10 mM dNTPs (133 μM), 1.5 μl each of forward and reverse primers (10 pmol), and 2.5 μl of template genomic DNA (75 ng), 0.15 μl of Taq DNA polymerase (0.75 U) Vivantis Technologies) in a final reaction volume of 15 μl. The PCR reaction profile was: DNA denaturation at 94°C for 5 min. followed by 35 cycles of 94°C for 1 min., 55°C for 1 min., 72°C for 1 min. and finally, 72°C for a final extension of 7 min. Re-screening of primers that did not amplify at these conditions was done by decreasing the annealing temperature sequentially by 1°C, and for the primers producing multiple bands, by increasing the annealing temperature by 1°C. The optimized SSR primers were then used for PCR amplification in eight varieties of pigeonpea. The PCR products were separated by electrophoresis in 4% Metaphor agarose gels (Lonza, Rockland ME USA) containing 0.1 μg/ml ethidium bromide in 1x TBE buffer at 130 V for 4 h. After electrophoresis, PCR products were visualized and photographed using gel documentation system Fluorchem™ 5500 (Alfa Innotech Crop., USA). The TSA sequences containing 550 SSRs were used for gene prediction using gene finding software MolQuest (FGENESH+) [57]. The position of SSRs was then analyzed for their exact location in the gene with respect to the open reading frame. To annotate the putative functions of the genes containing 550 validated SSRs, their unigene sequences were compared by BLASTX tool of NCBI at a cutoff bit score of 50 against the non-redundant protein database.

### SSR marker scoring and data analysis

The genotype profiles produced by SSR markers were scored manually. Each allele was scored as present (1) or absent (0) for each of the SSR loci. A total of 550 genic-SSR markers giving consistent expected size products were used for genotyping eight pigeonpea varieties; and 20 highly polymorphic loci of these were used for the diversity analysis on 30 genotypes. Markers that produced expected size of amplicons (100-200 bp) were scored for variation in amplicon size and the data analyzed for PIC using the formula described by Botstein *et al. *[58].

$$\;\text{PIC}=\text{1}-\sum Pi^{2}$$

Where, *Pi *is the frequency of the *i*^th ^allele in the set of genotypes analyzed, calculated for each SSR locus. The genetic similarity between any two genotypes was estimated based on Jaccard's similarity coefficient. All the 30 genotypes were clustered with the UPGMA analysis and SAHN procedure of the NTSYS-PC v2.10t [59].

## Authors' contributions

SD carried out RNA work, SSR mining and drafted the manuscript. SD, GK and BPS performed genotyping of SSR markers. DKG and VD carried out analysis of data generated by 454 GS-FLX sequencing. RR assembled the genotype set and provided plant materials. NKS in consultation with TRS and KG conceptualized the study, designed experiments and coordinated the study. GS-FLX sequencing and Newbler assembly was outsourced from Roche, Germany. GK, SS, SD, RR, MNS, BF, PK, RKV and DRC participated in drafting the manuscript. NKS finalized the manuscript. All authors read and approved the final manuscript.

## Supplementary Material

Additional file 1 **Frequency distribution of the pigeonpea genic-SSR of different sizes**. **a**. Unit length; **b**. Number of repeats; **c**. SSR length. Click here for file

Additional file 2 **Frequency distribution of SSR loci with different repeat motifs and number of repeats in the pigeonpea EST unigene contigs**. *Other 197 type of motifs out of total 207 motifs found in the pigeonpea transcriptome consisted of varied combinations. S. no. 1-10 are most frequently occurring motifs. Click here for file

Additional file 3 **Details of 2877 genic-SSR markers in pigeonpea**. The SSR motif, number of repeats, sequence of forward and reverse primers, annealing temperature and expected product size (bp) is indicated Click here for file

Additional file 4 **Details of 550 validated pigeonpea genic-SSR markers and predicted function of their genes based on BLASTX search results**. Sequence of forward and reverse primers, SSR repeat motifs, annealing temperature, expected allele size (bp) and putative gene function are indicated Click here for file

Additional file 5 ***Cajanus cajan *cultivars and wild relative species used for the validation and genetic diversity study using genic-SSR**. *Interspecific derivative involving *C. scarabaeoides*; ** Interspecific derivative involving *C. cajanifolius; *SD-Short duration; MD- Medium duration; LD-Long duration; PR-Perennial Click here for file
