# Supplementary material for: Development of genic-SSR markers by deep transcriptome sequencing in pigeonpea [Cajanus cajan (L.) Millspaugh]
Source: BMC Plant Biol. 2011 Jan 20;11:17. doi: 10.1186/1471-2229-11-17 (PMC3036606; doi:10.1186/1471-2229-11-17)
Supplement: Additional file 1 — Frequency distribution of the pigeonpea genic-SSR of different sizes. a. Unit length; b. Number of repeats; c. SSR length. [file 1471-2229-11-17-S1.DOC]

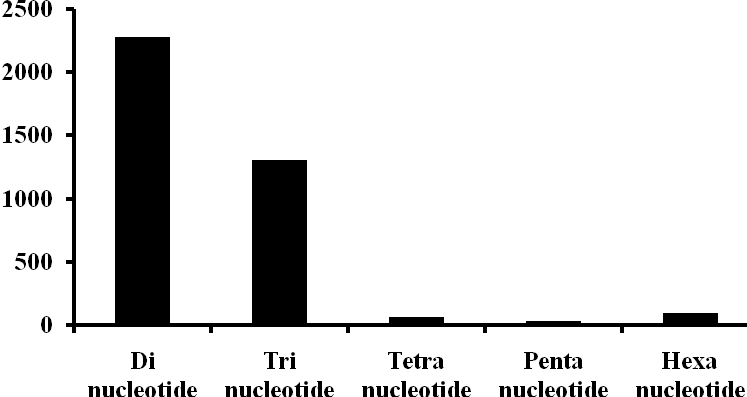


**a**


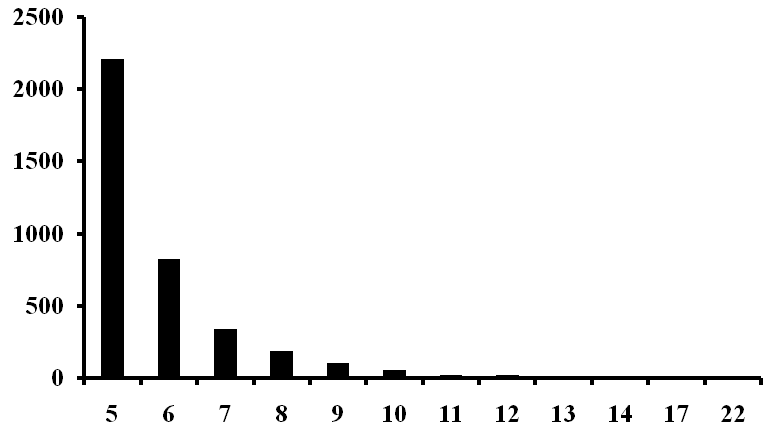


**b**


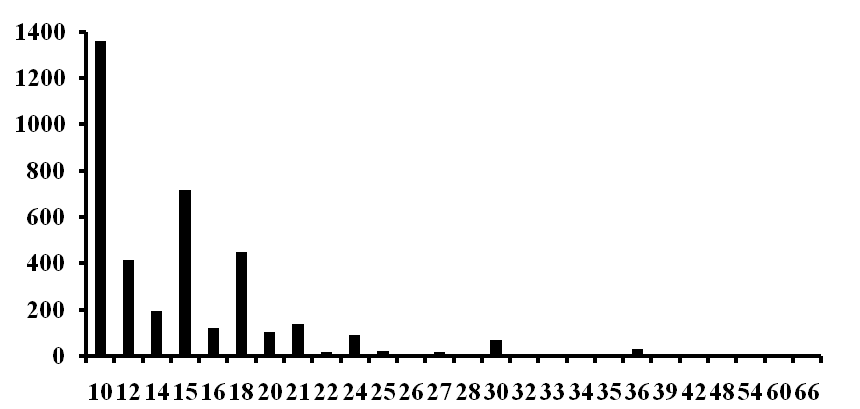


**c**

**Additional file 1 Frequency distribution of the pigeonpea genic-SSR** a**.** Unit length; b**.** Number of repeats; **c.**  SSR length
