# Supplementary material for: Development of genic-SSR markers by deep transcriptome sequencing in pigeonpea [Cajanus cajan (L.) Millspaugh]
Source: BMC Plant Biol. 2011 Jan 20;11:17. doi: 10.1186/1471-2229-11-17 (PMC3036606; doi:10.1186/1471-2229-11-17)
Supplement: Additional file 2 — Frequency distribution of SSR loci with different repeat motifs and number of repeats in the pigeonpea EST unigene contigs. *Other 197 type of motifs out of total 207 motifs found in the pigeonpea transcriptome consisted of varied combinations. S. no. 1-10 are most frequently occurring motifs. [file 1471-2229-11-17-S2.DOC]

**Additional file 2 Frequency distribution of the 10 most frequent SSR repeat motifs in the pigeonpea transcriptome with different levels of reiteration**

| S. no. | Repeat | Number of reiterations of the motif | | | | | | | | | | | Total |
| --- | --- | --- | --- | --- | --- | --- | --- | --- | --- | --- | --- | --- | --- |
|  | motif | 5 | 6 | 7 | 8 | 9 | 10 | 11 | 12 | 13 | 14 | 15+ |
| 1 | TC/GA | 362 | 111 | 65 | 44 | 24 | 20 | 10 | 5 | 2 | 2 | 1 | 646 |
| 2 | AG/CT | 328 | 128 | 58 | 32 | 39 | 24 | 4 | 10 | 3 | 2 | 1 | 629 |
| 3 | TA/TA | 242 | 58 | 25 | 18 | 7 | 2 | 3 | - | - | - | - | 355 |
| 4 | AT/AT | 208 | 58 | 25 | 18 | 7 | 2 | 3 | - | - | - | - | 321 |
| 5 | GAA/TTC | 85 | 37 | 8 | 12 | 3 | 1 | - | - | - | - | - | 146 |
| 6 | AC/GT | 76 | 25 | 10 | 3 | 3 | 1 | - | - | - | - | - | 118 |
| 7 | CTT/AAG | 46 | 32 | 10 | 6 | 4 | - | 1 | - | 1 | - | - | 100 |
| 8 | AGA/TCT | 24 | 17 | 6 | 3 | 1 | - | - | - | - | - | - | 51 |
| 9 | AAC/GTT | 22 | 20 | 7 | 1 | - | - | - | - | 1 | - | - | 51 |
| 10 | GAT/ATC | 28 | 16 | 2 | 4 | - | - | - | - | - | - | - | 50 |
|  | Other motifs* | 776 | 318 | 131 | 50 | 15 | 8 | 2 | 3 | - | - | 1 | 1304 |
|  | TOTAL | 2197 | 820 | 347 | 191 | 103 | 58 | 23 | 18 | 7 | 4 | 3 | 3771 |

*Additional 197 types of repeat motifs in the pigeonpea transcriptome with frequency less than 50 each
