# Supplementary material for: Development of genic-SSR markers by deep transcriptome sequencing in pigeonpea [Cajanus cajan (L.) Millspaugh]
Source: BMC Plant Biol. 2011 Jan 20;11:17. doi: 10.1186/1471-2229-11-17 (PMC3036606; doi:10.1186/1471-2229-11-17)
Supplement: Additional file 5 — Cajanus cajan cultivars and wild relative species used for the validation and genetic diversity study using genic-SSR. *Interspecific derivative involving C. scarabaeoides; ** Interspecific derivative involving C. cajanifolius; SD-Short duration; MD- Medium duration; LD-Long duration; PR-Perennial [file 1471-2229-11-17-S5.DOCX]

**Additional file 5– *Cajanus Cajan* cultivars and wild relative species used for the validation and genetic diversity study using genic-SSR**

| **S. No** | **Pigeonpea**  **genotype** | **Species** | **Gene pool** | **Maturity group** | **Source** |
| --- | --- | --- | --- | --- | --- |
| 1 | Asha | *C. cajan* | Primary | MD | ICRISAT, Hyderabad |
| 2 | UPAS 120 | *C. cajan* | Primary | SD | IIPR, Kanpur |
| 3 | HDM 04-1 | *C. cajan* | Primary | SD | CCSHAU, Hisar |
| 4 | Pusa Dwarf | *C. cajan* | Primary | SD | IARI, New Delhi |
| 5 | H2004-1 | *C. cajan* | Primary | SD | CCSHAU, Hisar |
| 6 | Bahar | *C. cajan* | Primary | LD | IIPR, Kanpur |
| 7 | Maruti | *C. cajan* | Primary | MD | IIPR, Kanpur |
| 8 | TTB7 | *C. cajan* | Primary | LD | IIPR, Kanpur |
| 9 | Pusa 992 | *C. cajan* | Primary | SD | IARI, New Delhi |
| 10 | PS-971 | *C .cajan* | Primary | SD | IARI, New Delhi |
| 11 | PS-956 | *C. cajan* | Primary | SD | IARI, New Delhi |
| 12 | Pusa-9 | *C. cajan* | Primary | LD | IARI, New Delhi |
| 13 | JA-4 | *C. cajan* | Primary | MD | JNKVV, Jabalpur, |
| 14 | Kudarat | *C. cajan* | Primary | LD | IARI, New Delhi |
| 15 | PCMF40* | *C. cajan* | Primary | SD | IARI, New Delhi |
| 16 | PCMF43-7* | *C. cajan* | Primary | SD | IARI, New Delhi |
| 17 | PCMF39-1* | *C. cajan* | Primary | SD | IARI, New Delhi |
| 18 | GT288A(CMS)* | *C. cajan* | Primary | MD | GAU, S. K. Nagar |
| 19 | GTR-9 | *C. cajan* | Primary | MD | GAU, S. K. Nagar |
| 20 | GTR-11 | *C. cajan* | Primary | MD | GAU, S. K. Nagar |
| 21 | ICPA2089A(CMS)** | *C. cajan* | Primary | SD | ICRISAT, Hyderabad |
| 22 | ICPR2438 | *C. cajan* | Primary | SD | ICRISAT, Hyderabad |
| 23 | ICP15809 | *Rhynchosia aurea* | Tertiary | PR | IIPR, Kanpur |
| 24 | ICP15666 | *Cajanus platycarpus* | Tertiary | PR | IIPR, Kanpur |
| 25 | ICP15632 | *Cajanus cajanifolius* | Secondary | PR | IIPR, Kanpur |
| 26 | ICP16664 | *Cajanus platycarpus* | Tertiary | PR | IIPR, Kanpur |
| 27 | ICP15817 | *Rhynchosia bracteata* | Tertiary | PR | IIPR, Kanpur |
| 28 | ICPW95 | *Cajanus sericeus* | Secondary | PR | IARI, New Delhi |
| 29 | ICP15624 | *Cajanus albicans* | Secondary | PR | IIPR, Kanpur |
| 30 | ICP15642 | *Cajanus lineatus* | Secondary | PR | IIPR, Kanpur |

# *Inter-specific derivative involving *C. scarabaeoides*; ** Inter-specific derivative involving *C. cajanifolius;* SD-Short duration; MD- Medium duration; LD-Long duration; PR-Perennial
